# Evaluation of logistics management information system and availability of non-program tracer drugs in public health facilities in Bahir Dar City, Northwest Ethiopia

**DOI:** 10.1371/journal.pone.0302319

**Published:** 2024-04-18

**Authors:** Biset Asrade Mekonnen, Minichile Chanie Worku, Bereket Bahiru Tefera

**Affiliations:** 1 Department of Pharmacy, College of Medicine and Health Sciences, Bahir Dar University, Bahir Dar, Ethiopia; 2 Department of Pharmaceutical Analysis, School of Pharmacy, College of Medicine and Health Sciences, University of Gondar, Gondar, Ethiopia; University of Gondar, ETHIOPIA

## Abstract

The logistics management information system (LMIS) plays a crucial role in effective record-keeping and reporting, ensuring efficient management of stock status and consumption data. A proficient LMIS improves accountability and supports informed logistic decisions in healthcare. Conversely, a subpar LMIS negatively affects essential medicine availability, compromising overall healthcare service efficiency. This study aimed to evaluate the status of the logistics management information system and the availability of non-program tracer drugs (NPTDs) in public health facilities within Bahir Dar City. This study employed an institutional-based cross-sectional study. Data were collected from February 20 to April 30, 2022 in 12 public health facilities located in Bahir Dar City. Structured questionnaire and data abstraction formats were used to gather pertinent data. After checking for completeness, the data were analyzed using Microsoft Excel and SPSS version 23. The primary analytical outcomes involved descriptive statistics, encompassing frequencies, averages, and percentages, which were subsequently presented in tables and figures. Bin card and Internal Facility Report and Resupply Form were the only blank LMIS tools available in all health facilities, while stock-record card was the least available, present in only 2(16.7%) facilities. Nine health facilities (75%) used self-prepared forms to request NPTDs from the Ethiopian Pharmaceutical Supply Agency (EPSA) at the end of the review period, whereas 7(58.3%) used official letters for emergency orders. Additionally, seven health facilities (58.3%) used the Health Commodities Management Information System On the day of the visit, 78.68% of NPTDs were available. Tetracycline eye ointment 1% had the longest stock-out duration, lasting for a mean 69.64 days. Rather than using the RRF, most of facilities opted for their own forms to request NPTDs from EPSA. While it is advisable for all health facilities to maintain continuous availability of tracer drugs, this study revealed that the current state of non-program tracer drug availability falls short of meeting this expectation.

## Introduction

The Logistics Management Information System (LMIS) involves recording, processing, and using logistic information through either paper or computer-based systems [1–3]. A well-functioning LMIS plays a crucial role in collecting essential information on stock status and consumption, promoting accountability, cost-effectiveness, and efficient decision-making [3–5]. Precise record-keeping is crucial as it guides decisions on pharmaceutical selection, quantification, procurement, and utilization [1, 6]. For an LMIS to be effective, it requires an optimal blend of skilled personnel, efficient procedures, and suitable technologies that are sustainable and widely accepted by users at various levels [5, 7, 8]. The system is most impactful when adequately trained and competent personnel are involved in recording, analyzing, managing, and leveraging the data across all levels [1, 5].

LMIS should capture three essential data items: stock on hand, consumption data, and losses and adjustments, all of which play a crucial role in making informed logistics decisions [1, 5, 9]. In Ethiopia, these essential data are documented and reported through the Integrated Pharmaceuticals Logistics System (IPLS) using various forms, including the bin-card, stock-record card, Internal Report and Resupply Form (IFRRF), Report and Requisition Form (RRF), and Health Post Monthly Report and Resupply Form (HPMRRF) [1, 10, 11]. Despite these efforts, studies conducted in Ethiopia have identified significant challenges, indicating that most health facilities experience poor data quality in their stock-keeping records and reports [11].

In low-income countries, including Ethiopia, logistic managers often depend on experience, speculation, and insufficient information systems for decision-making [7, 11]. In cases where records are lacking, the pharmaceutical supply chain is susceptible to inaccurate information, leading to frequent stock-outs or overstocking, both of which incur significant costs [1, 3, 6]. The widespread implementation of LMIS across all levels of the supply chain can have a substantial impact on program effectiveness by ensuring product availability, enhancing community service-seeking behavior, and increasing professional satisfaction [4, 9, 12]. However, sustaining the LMIS is challenging because of scarce human and other resources for data analysis, validation, information exchange, and implementation, compounded by inadequate government support for system maintenance and upgrades [1, 6, 9].

A study conducted in Oromia revealed that hospitals maintained updated bin cards for 43.8% of their products, whereas health centers and health posts had updated bin cards for 32.9% and 32% of their products, respectively [13]. A study conducted in the Southern Nations, Nationalities, and Peoples’ Region (SNNPRS) of Ethiopia indicated that merely 60% of healthcare facilities possessed comprehensive RRFs [14]. A study conducted in the Amhara region also indicated that the average accuracy of stock-keeping records was 56.7% [15]. Another study conducted in the Amhara Regional State reported that only 52.5% of health facilities used a bin card, while 49.2% did not use a stock record card.

In Ethiopia, although studies have evaluated the LMIS practices of program medicines, there is a gap in understanding the practices for non-program essential medicines, especially in the study area. Consequently, this study aims to evaluate the LMIS practices and availability of non-program tracer drugs (NPTDs) in public health facilities located in Bahir Dar City. To the best of our knowledge, this study marks the first attempt to assess LMIS practices exclusively for non-program medicines in this specific study area. Therefore, this research can act as a foundational step for future studies related to the management of non-program medicines.

## Methods and materials

### Study area and period

This study was conducted in Bahir Dar City Administration, one of the special zones in the Amhara Region. Bahir Dar, the capital city of the Amhara Regional State, is situated 578 kilometers northwest of Addis Ababa. The projected total population of Bahir Dar in 2022, extrapolated from the 2014 national census conducted by the Central Statistical Agency of Ethiopia, stood at 455,901 residents [16]. Within Bahir Dar city, there are 12 public health facilities, comprising two public hospitals and ten public health centers. The study gathered data from different public health facilities from February 20 to April 30, 2022.

### Study design

An institution-based cross-sectional study design was implemented to assess the logistic management information system and the availability of non-program tracer drugs in public health facilities in Bahir Dar City.

### Study population

The study population comprised public health facilities and healthcare professionals in Bahir Dar City. Additionally, various documents, including bin-cards, stock-record cards, standard operating procedure of IPLS, IFRR, and RRF, were considered part of the study population.

### Inclusion and exclusion criteria

The pharmaceutical store managers in the included public health facilities, with a tenure exceeding 6 months in their respective facilities, were included in the study. Bin-cards, stock-recording cards, standard operating procedure of IPLS, IFRRs, and RRFs were the documents included. Documents without complete information, as well as program medicines, were excluded from the study.

### Sample size determination and sampling procedure

There are twelve public health facilities in the Bahir Dar City Administration, consisting of two hospitals and ten health centers. Consequently, the study incorporated all ten health centers and two hospitals within the city. Tracer drugs, designated by the Federal Ministry of Health of Ethiopia to assess the availability of essential medicines in public health institutions, were employed in this study [17]. Out of the 11 tracer drugs listed, 5 of them were non-program tracer drugs and chosen to be included in this study. The documents subjected to review included bin-cards, stock-recording cards, the standard operating procedure of IPLS, IFRRs, and RRFs. In this study, we used one RRF and five bincards from each facility. This approach resulted in 12 RRFs and 60 bin-cards being included in the study for document review.

Purposive sampling was used to select pharmacy store managers from all health facilities to participate in the survey, considering their roles in overseeing the overall activities of pharmaceutical logistics, making them capable of providing the necessary information. Consequently, the study incorporated 12 pharmacy store managers.

### Outcome variable

The outcome variables of this study were the implementation status of LMIS and the availability of NPTDs in public health facilities in Bahir Dar.

### Data collection instruments

A structured questionnaire and data abstraction format adapted from the Logistics Indicator Assessment Tool (LIAT) were used to collect data regarding the status of logistics management information system practices and the availability of NPTDs in public health facilities [18]. The structured questionnaire comprised two sections. The first section contained questions aimed at assessing the profile of health facilities and healthcare professionals included in the study. The second section included crucial questions to capture data related to the status of LMIS practices (availability, utilization, updating, and functionality of LMIS tools). The data abstraction format was used to gather data related to product availability and stockout duration.

### Data collection procedures

Each public health facility was assigned a pharmacy professional data collector who underwent training in the data-gathering process. The collection of data involved the use of structured questionnaires and document reviews, which included bin-cards, stock-recording cards, IFRRs, RRFs, and the standard operating procedure of IPLS. Information regarding drug availability on the day of the visit was obtained through physical counts. To assess frequency and stock-out duration, we reviewed bin-card transactions of the last 6 months before study period. The principal investigator supervised the data collection process and reviewed the completed questionnaires to check for any inconsistencies.

### Data quality assurance

To ensure data quality, the questionnaires were meticulously designed and subjected to pretesting. A pretest, involving 5% of the sample size, was performed, specifically at a health center in the south Gondar zone. This step validated the clarity and comprehensibility of the prepared questionnaires and abstraction formats, thereby ensuring the reliability of the survey instrument. Following the pilot test, adjustments were made to the tools based on the feedback received. Cronbach’s alpha for the test was also determined to be 0.74, indicating a satisfactory level of internal consistency. Additionally, the principal investigator scrutinized the completeness of the data to ensure accurate and appropriate filling.

### Data processing and analysis

Before initiating the analysis, the completeness of the data gathered from the questionnaires was carefully reviewed. Subsequently, the data were entered and analyzed using Statistical Package for Social Science (SPSS) version 23 and Microsoft Excel 2010. The primary outputs for the analysis were descriptive statistics, encompassing frequencies, averages, and percentages. The findings were then presented in tables and graphs for clarity.

### Operational definition

*Tracer Drugs*: These are medications designated by the Federal Ministry of Health in Ethiopia to be consistently stocked and available at all government health institutions every day, around the clock, and throughout the year.

*Non-program drugs*: Medications that fall under the management of the Revolving Drug Fund, where charges are levied for the provision of medicines in health facilities.

*Non-program tracer drugs*: Refers to tracer drugs that are not included in the category of program drugs.

*Blank Bin Card*: An unused bin card that has been prepared and is ready for future use.

*Bin Card Utilization*: We consider a facility as utilizing bin cards if there is an assigned bin card for all five NPTDs at the health facility on the day of data collection.

*Regularly Updated Bin Card*: A bin card is deemed regularly updated if the bincard of NPTD at a health facility has recorded transactions for at least the preceding 30 days before the study period and is in accordance with the physical inventory on the specific day of data collection.

### Ethical considerations

According to the Ethiopian National Research Ethical Review Guidelines, a study investigating government programs that provide public goods or services is exempt from ethical review if the data are collected in a way that makes it impossible to identify the study participants [19]. Moreover, before engaging in the study, participants were verbally asked to give their consent. Additionally, the initial question in the questionnaire was designed to inquire about the participant’s willingness to take part in the data collection. Before granting consent, the participants were informed about the study objectives and what was expected of them. They were also assured of their right to withdraw at any point. The decision to use verbal informed consent aligns with the National Research Ethical Review Guidelines [19], which consider such an approach acceptable for studies with minimal risks. Participants were also emphasized on the confidentiality of the information collected during the study.

## Result

Among the types of health facilities examined in this study, the majority consisted of health centers, comprising 83.3% of the sampled facilities. More than half of the health facilities (58.3%) implemented both the Health Commodities Management Information System (HCMIS) and paper-based IPLS. Personnel qualification of participants showed that 75% of them were pharmacists. On average, participants held the position of pharmacy storekeeper for 1.7 years within their respective health facilities (Table 1).

**Table 1:** Attributes of health facilities and health professionals participated in the study.

		Frequency (N = 12)	Percentage (%)
Type of health facilities	Hospital	2	16.7
	Health center	10	83.3
Type of IPLS implementation	Paper-based IPLS	5	41.7
	Both HCMIS and paper-based IPLS	7	58.3
Qualification of Personnel	Druggist	3	25
	Pharmacist	9	75

### Availability of blank logistics management information system formats

All public health facilities (100%) had blank bin-cards and IFRR forms in their pharmacy stores. However, the stock-record card was the least available LMIS form, present in only 2 (16.7%) of the health facilities (Table 2).

**Table 2:** Availability of blank IPLS’s LMIS formats in health facilities (N = 12).

LMIS Forms	Available	Not-Available
	{n (%)}	{n (%)}
IPLS SOP	10(83.3%)	2(16.7%)
Bin-card	12(100%)	0
Stock record card	2(16.7%)	10(83.3%)
RRF	11(91.7%)	1(8.3%)
IFRR	12(100%)	0

### Utilization and regular updating status of bin-cards

All bin-cards in use were consistently maintained up-to-date. Specifically, bin-cards for amoxicillin 500mg capsules were regularly updated in all health facilities, with a utilization rate of 100%. However, 25% of the health facilities did not use a bin-card for oral rehydration salt (Table 3).

**Table 3:** Percentage of health facilities where bin-cards are utilized and regularly updated, (n = 12).

Tracer drugs	Utilization of bin-card	Regularly updated bin-cards
	n(%)	n(%)
Amoxicillin 500mg capsule	12(100)	12(100)
Oral rehydration salt	9(75)	9(75)
Mebendazole 100mg tablet	11(91.7)	11(91.7)
Tetracycline eye ointment 1%	11(91.7)	11(91.7)
Paracetamol 500mg table	11(91.7)	11(91.7)

### LMIS forms used to request non-program tracer drugs

Under normal conditions, only 2(16.7%) of the facilities used RRF to request non-program tracer drugs from Ethiopian Pharmaceutical Supply Agency(EPSA) at the end of the review period. Conversely, 9(75%) health facilities used forms they had prepared themselves for this purpose. In emergency situations, 2(16.7%) health facilities utilized RRF, while 7 (58.3%) utilized official letters for ordering (Table 4).

**Table 4:** Formats used by health facilities to request NPTDs from EPSA (N = 12).

Types of forms	For orders at the end of the review period	For emergency ordering
	n(%)	n(%)
IFRR	1(8.3%)	0
RRF	2(16.7%)	2(16.7%)
Facility’s own form	9(75%	3(25%)
Letter	0	7(58.3%)
Orally	0	0
Others	0	0

### Frequency of emergency orders placed by health facilities

**Fig 1:**
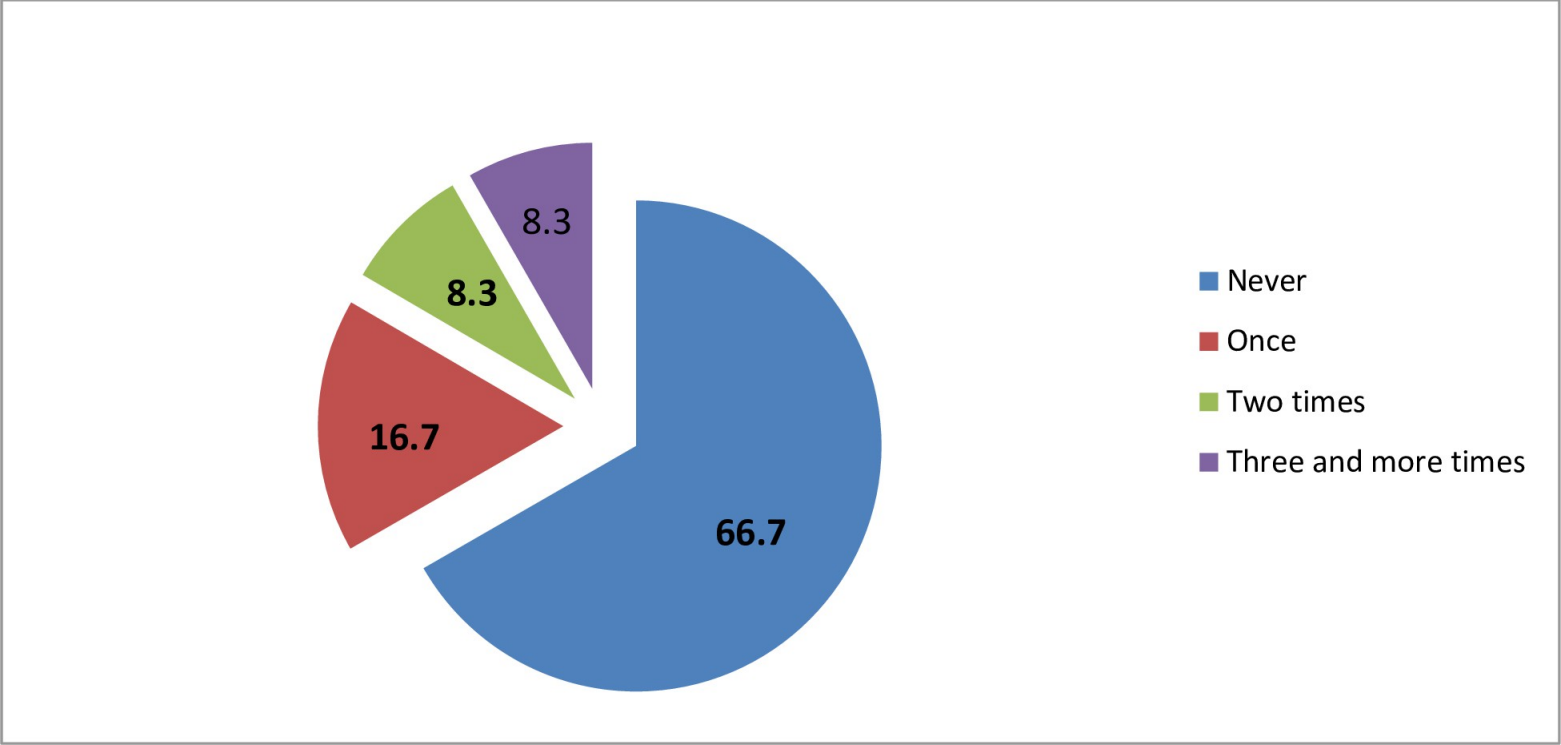
Percentage of health facilities with a frequency of emergency orders in the last 3 months before the study period (n = 12).

The majority of health facilities, 8(66.7%), did not place emergency orders for non-program tracer drugs during the last 3 months before the study period. Conversely, one facility (8.3%) initiated more than three emergency orders drugs during this period (Fig 1).

### Availability, functional status, and application of HCMIS

More than half of the health facilities (58.3%) used HCMIS to manage pharmaceuticals in their stores and all of them were functional. Of these facilities, two were hospitals, while the remaining five (71.4%) were health centers (Table 5).

**Table 5:** Availability and functional status of HCMIS in health facilities.

Name of health facilities	Available	Functional (on the day of the visit)
	Yes/No	Yes/No
Felege Hiwot comprehensive specialized hospital	Yes	Yes
Addis Alem primary hospital	Yes	Yes
Shembit health center	No	N/A
Bahir Dar health center	Yes	Yes
Han health center	Yes	Yes
Shumabo health center	No	N/A
Ginbot 20 health center	No	N/A
Abay health center	No	N/A
Zegie health center	No	N/A
Tisabay health center	Yes	Yes
Meshentie health center	Yes	Yes
Zenzelema health center	Yes	Yes

**Fig 2:**
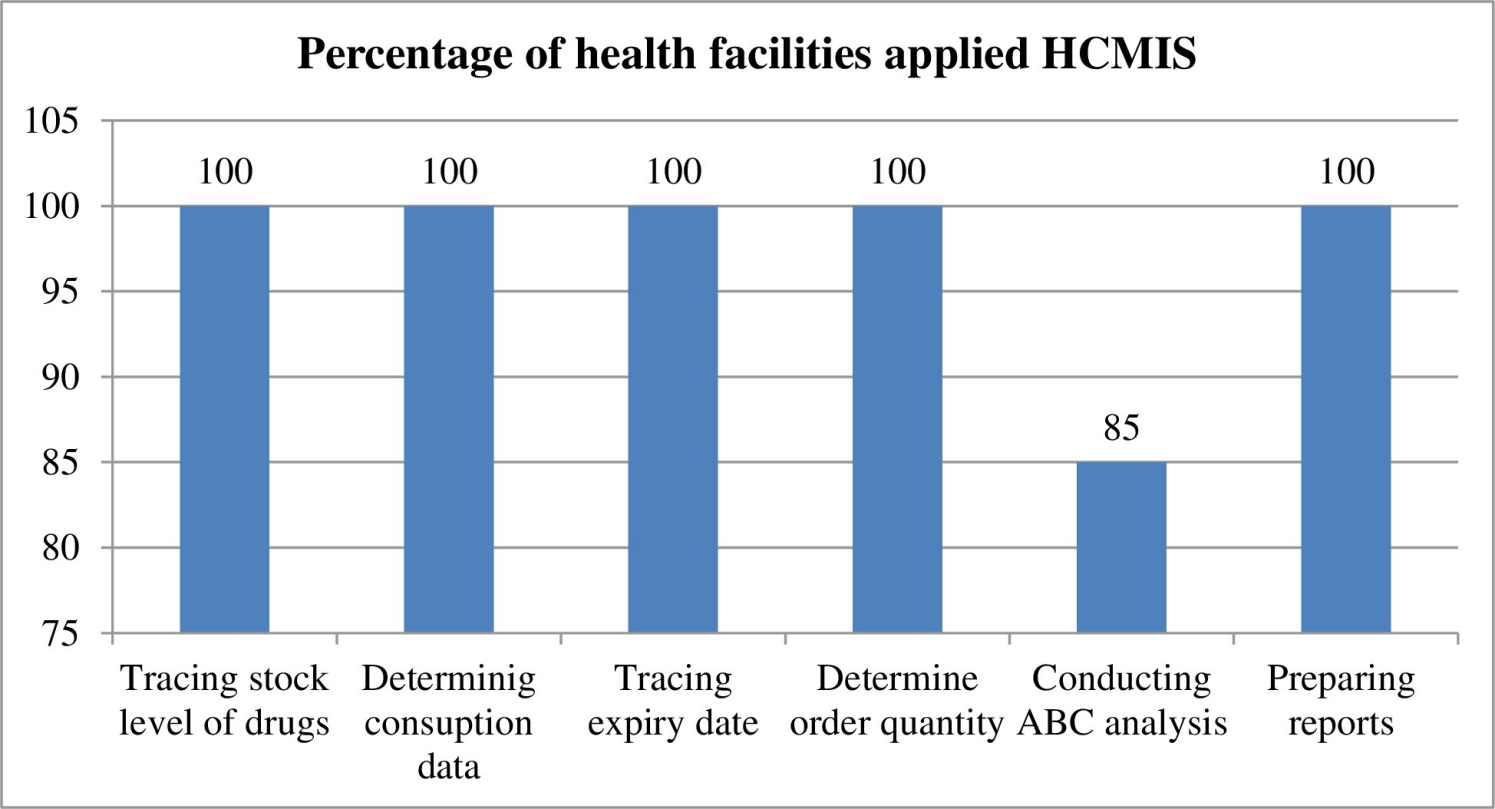
Percentage of health facilities that applied HCMIS for different decisions.

Among the health facilities that have HCMIS, 100% of them used it for following tasks such as tracing stock levels, determining consumption, tracing expiration dates, determining issue quantities, determining order quantities, and preparing reports. Moreover, 85.7% of the health facilities used it for conducting ABC analysis (Fig 2).

### Availability of non-program tracer drugs on the day of the visit

This study revealed that the overall availability of NPTDs on the day of the visit was 78.68%. Among all NPTDs, only amoxicillin 500mg capsule was available in all health facilities (Table 6).

**Table 6:** Percentage of health facilities where NPTDs are available on the day of the visit (n = 12).

Tracer drugs	Frequency (N = 12)	Percentage (%)
Amoxicillin 500 mg capsule	12	100
Oral rehydration salt	6	50
Mebendazole 100mg tablet	11	91.7
Tetracycline eye ointment 1%	6	50
Paracetamol 500mg tablet	11	91.7
**Average**		**78.68**

### Frequency and length of stock-out duration

This study revealed that in most of the health facilities, 9(75%), tetracycline eye ointment 1% was out of stock. In contrast, amoxicillin 500mg capsules and paracetamol 500mg tablet were only out of stock in one health facility. Over the last 6 months, tetracycline eye ointment 1% had the highest mean stock-out frequency of 0.82 (SD = 0.405) and had a longer average stock-out duration compared to other NPTDs (mean = 69.64 days, SD = 59.897). On the other hand, paracetamol 500mg tablets had the shortest stock-out duration, with an average of 0.09 days (SD = 0.302) (Table 7).

**Table 7:** Descriptive statistics related to stock-out of non-program tracer drugs in the last 6 months before the study period.

Tracer Drugs	Frequency of health facilities encountered stock-out (N = 12)	Frequency of stock-outs	Stock -out days
	n(%)	Mean±SD	Mean±SD
Amoxicillin 500 mg capsule	1(8.33)	0.08±0.289	1.58±5.485
Oral rehydration salt	6(50)	0.42±0.441	33.33±67.825
Mebendazole 100mg tablet	3(25)	0.18±0.405	2.27±5.179
Tetracycline eye ointment 1%	9(75)	0.82±0.405	69.64±59.897
Paracetamol 500mg tablet	1(8.33)	0.09±0.302	0.09±0.302

## Discussion

Blank bincards and IFRR forms were present in every health facility. This aligns with the Ethiopian Hospitals Reform Implementation Guidelines (EHRIG) standard, which recommends a 100% presence [20]. Among IPLS’s LMIS forms, the stock-recording card was the least available, found in only two health facilities. Similar findings were reported in studies conducted in Dessie and East Gojjame Zone, where bincards, IFRR, and RRF were available in every health facility, whereas the stock-recording card was the least available [3, 11]. On the other hand, another study in Ethiopia reported that bin-cards and RRF were available in all facilities, but IFRR was available in only 83% [13]. It is crucial for every blank logistic record to be consistently available when needed [20]; nevertheless, this study identified some shortfalls.

All bincards used in all health facilities were regularly updated, showing a significantly higher rate than studies conducted in Dessie and West Gojjam Zone, which reported rates of 89% and 63.3%, respectively [3, 11]. This difference may be attributed to the smaller sample size in the current study, as a larger sample size could encompass facilities with poorer implementation, potentially impacting the overall performance assessment of the study. All health facilities consistently maintained updated bin-cards for amoxicillin 500mg capsules; however, oral rehydration salt had the least updated bin-cards, with updates recorded in only 75% of the facilities. Likewise, a study in Dessie found that oral rehydration salt had the least frequently updated bin-card [3]. Bin-cards play a crucial role in drug management by providing essential information on stock balance, receipt and issue quantities, and expiration dates [10, 21].

The SOP for IPLS recommends that health facilities should complete RRF as per their review period and collect products from the affiliated EPSA branch [10, 22]. However, the results indicate that only 16.7% of the health facilities used RRF, while the majority used their own forms for reporting and requesting these drugs. Furthermore, 8.3% of the facilities used IFRR for requesting medicines from EPSA; this form is only used for distributing medicines from the health facility’s medicine store to dispensing units [22]. This finding could be attributed to the fact that it may be easier for pharmacists to create their own format than to fill out the RRF’s relatively large number of columns. These findings indicate that even though IPLS was designed to manage both program and non-program medicines in the same way, health facilities are not applying IPLS for requesting non-program medicines from EPSA in a similar way to program medicines.

Seven health facilities (58.3%) implemented HCMIS in their stores, utilizing it for data gathering and analysis to inform various decisions. This outcome marked a significant improvement compared with studies conducted in Dessie and Addis Ababa, which reported rates of 33.33% and 32.4%, respectively [3, 23]. Nevertheless, all findings indicate that the automated logistics management information system in Ethiopia is still in the early stages of development. In addition, these studies also reported that all available HCMIS are fully functional. When used effectively, automation simplifies and speeds up complex tasks, increases accuracy, generates timely reports, and provides management information for decision-making [21, 23].

Ideally, maintaining a 100% availability of tracer drugs in all health facilities is recommended [15]. However, this study revealed that the availability of non-program tracer drugs on the day of the visit was 78.68%. This low availability may be attributed to the poor implementation of LMIS and suboptimal inventory management practices. However, this finding is higher than a report from a study conducted in Kenya and Ghana, which reported 68.7% and 64.1%, respectively [24, 25]. This finding is consistent with reports from Ethiopian studies [3, 15, 26] and slightly better than a report from another Ethiopian study that found 70.16% [27]. However, it is lower than the findings of the national survey of the IPLS, which claimed 89% availability [28], and another study conducted in Ethiopia that reported 86.5% availability [29]. In general, the findings of this study indicate that the availability of TDs falls significantly below the WHO-recommended target of 100% [30].

The NPTD that experienced stock-outs (at least once) in most health facilities during the last six months before the survey was tetracycline eye ointment 1%, with 75% of the health facilities reporting stock-outs. This study also found that tetracycline eye ointment 1% had the highest mean stock-out frequency and the longest duration of stock-out (69.64 days). Similarly, a study carried out in the Amhara region indicated that tetracycline eye onitment 1% had the longest stock-out duration, lasting for 127 days [29]. This highlights the need to enhance the availability of essential medicines in health facilities. In comparison, paracetamol 500mg tablets and amoxicillin 500 mg capsules had the lowest mean stock-out frequency and stock-out duration.

### Study limitations

The investigation omitted an exploration into the reasons behind the suboptimal LMIS practices and the availability of non-program tracer drugs in health facilities. Furthermore, the study didn’t address the examination of critical indicators such as timeliness, completeness, and accuracy of logistics-related data, which are essential for assessing the quality of the LMIS system.

## Conclusion

Our study provides valuable insights into the logistics management practices within health facilities in Bahir Dar City, Ethiopia. Despite some adherence to standard guidelines, such as the presence of blank bin-cards and IFRR forms in all facilities, there are some deficiencies in LMIS implementation, such as the utilization of incorrect formats for requesting medicines. Notably, while there is room for improvement, the use of automated systems like HCMIS shows promising progress. However, challenges persist, as evidenced by the suboptimal availability of tracer drugs and frequent stock-outs of essential medicines like tetracycline eye ointment 1%. Addressing these shortcomings is imperative to ensure the effective and efficient delivery of pharmaceutical services and improve overall healthcare outcomes in Ethiopia. This study has notable limitations; hence, future researchers are advised to consider them when conducting studies in this area.

## Supporting information

S1 Appendix (XLSX)

S1 Table Availability of blanck LMIS forms during the day of visit. (XLSX)

S2 Table Utilization and regualr updating status of bincard for each tracer drugs at each health facilities. (XLSX)

S3 Table Forms used by health facilities to request NPTDs. (XLSX)

S4 Table Frequency of emergency orders placed by health facilities 3 months before dta collection. (XLSX)

S5 Table Availability, and functional status of HCMIS. (XLSX)

S6 Table Application of HCMIS at each health facilities. (XLSX)

S7 Table Availability of non-program tracer drugs on the day of the visit. (XLSX)

S8 Table Stock-out during the last 6 months. (XLSX)
